# Comparison of Extended Arch Versus Hemiarch Replacement in Elderly Patients With Type A Aortic Dissection: The Shizuoka Kokuho Database

**DOI:** 10.1093/icvts/ivag017

**Published:** 2026-01-10

**Authors:** Daisuke Arima, Yoko Sato, Yoshihiro Tanaka

**Affiliations:** Shizuoka Graduate School of Public Health, Shizuoka Graduate University of Public Health, Shizuoka, Shizuoka 420-0881, Japan; Department of Cardiovascular Surgery, Fujieda Municipal General Hospital, Shizuoka, 426-8677, Japan; Shizuoka Graduate School of Public Health, Shizuoka Graduate University of Public Health, Shizuoka, Shizuoka 420-0881, Japan; Shizuoka Graduate School of Public Health, Shizuoka Graduate University of Public Health, Shizuoka, Shizuoka 420-0881, Japan

**Keywords:** aorta, surgery, real-world data

## Abstract

**Objectives:**

Extended arch replacement (EAR) and hemiarch replacement (HAR) are surgical options for type A acute aortic dissection (AAD). However, the effectiveness of EAR compared with HAR, particularly in elderly patients, remains unclear owing to its invasiveness and complications. This study aimed to compare the postoperative outcomes of EAR and HAR in elderly patients with type A AAD.

**Methods:**

This retrospective cohort study used data from the Shizuoka Kokuho Database, a prefecture-wide, multi-institutional administrative claims database managed by the Shizuoka Prefectural Government. We identified patients aged ≥60 years with type A AAD who underwent HAR or EAR between April 2012 and September 2022. Propensity score matching (PSM) was employed to balance the baseline characteristics between the groups. The primary outcome was all-cause mortality. The secondary outcome included the incidence of reoperation for bleeding.

**Results:**

A total of 774 patients were included (174 undergoing EAR and 600 undergoing HAR). After PSM, 167 matched pairs were analysed. Kaplan-Meier curves revealed no significant differences in survival between both procedures (log-rank test, *P* = .739). Cox proportional hazards analysis also revealed no significant differences in all-cause mortality between the EAR and HAR groups (hazard ratio: 1.08, 95% confidence interval: 0.70-1.66). However, the incidence of reoperation for bleeding was higher in the EAR group than in the HAR group (20 [12.0%] vs 7 [4.2%], *P* = .012).

**Conclusions:**

Although no statistically significant difference in postoperative mortality was observed between EAR and HAR, the incidence of reoperation for bleeding was higher in the EAR group. Therefore, the indication for EAR in elderly patients with type A AAD should be considered with caution.

## Graphical Abstract

**Figure:**
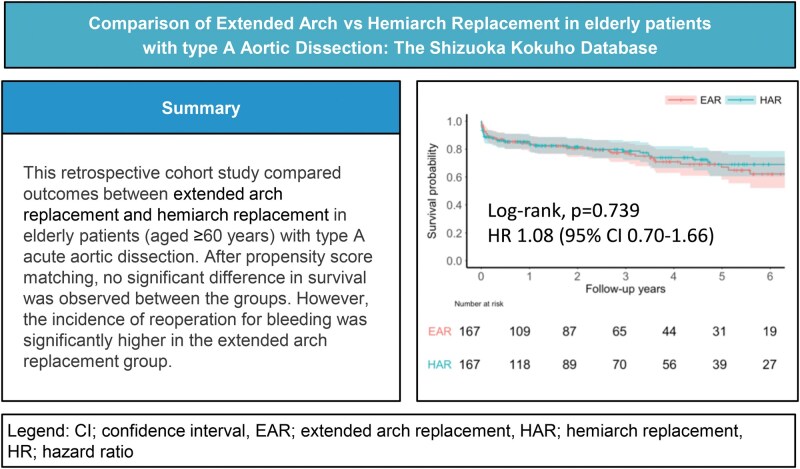

## INTRODUCTION

Acute aortic dissection (AAD) is a life-threatening condition characterized by tears in the aortic wall that lead to high morbidity and mortality rates. Extended arch replacement (EAR), which involves the reconstruction of the neck vessels, is increasingly being performed, especially in younger patients, owing to the potential for improved long-term outcomes.^1^ Hemiarch replacement (HAR) is frequently performed as a life-saving procedure. Some previous studies have reported conflicting results regarding EAR for type AAD, with one showing increased mortality while others found no significant difference.^2^^,^^3^ Elderly patients are more likely to have comorbidities such as myocardial infarction, diabetes, chronic lung disease, and hypertension, which are recognized prognostic factors for poorer outcomes.^4^ In this population, highly invasive surgical procedures are often avoided when possible. The effectiveness of EAR compared with HAR in elderly patients with type A AAD remains unclear. Therefore, this study aimed to compare the postoperative outcomes of HAR and EAR in elderly patients with type A AAD (aged ≥60 years) using real-world data.

## PATIENTS AND METHODS

### Study design and data source

The study protocol was conducted in accordance with the ethical principles outlined in the Declaration of Helsinki and approved by the ethics committee of the institutional review ethics board (SGUPH_2021_001_069), and informed consent was not required due to the retrospective nature of the study and the use of anonymized data.

This retrospective cohort study was conducted using the Shizuoka Kokuho Database (SKDB), a prefecture-wide, multi-institutional administrative claims database managed by the Shizuoka Prefectural Government. The SKDB includes 2 654 305 residents enrolled in National Health Insurance and the Latter-Stage Elderly Healthcare System in Shizuoka Prefecture, Japan. The current version of the SKDB covers the period from April 2012 to September 2022 (SKDB version 2024.1, Analysis Data Generation System version 4.0). The National Health Insurance primarily covers self-employed individuals, retirees, and others not covered by employer-based insurance, while the Latter-Stage Elderly Healthcare System provides coverage for individuals aged ≥75 years. Most individuals transition out of employee health insurance by age 65 years and join the National Health Insurance; at the age of 75 years, almost all individuals shift to the Latter-Stage Elderly Healthcare System.

The SKDB contains individual-level data on age, sex, observation period, monthly claims for diagnosis, procedures, medications, and reasons for withdrawal, including death. Data on surgical procedures were extracted from the database using billing codes. Similarly, medications and diagnoses were identified based on billing codes corresponding to the Anatomical Therapeutic Chemical Classification System and the International Classification of Diseases, 10th Revision, respectively. Previous study validated the reliability and research quality of the SKDB.^5^

### Study population and participants

The study period was between April 2012 and September 2022. We identified patients aged ≥60 years with type A AAD who underwent artificial graft replacement based on monthly procedural claims recorded in the SKDB. To define type A AAD, we extracted cases using the following the health insurance claim diagnoses; “Stanford A, acute aortic dissection,” “DeBakey I, acute aortic dissection,” and “DeBakey II, acute aortic dissection.”

EAR involves partial or total arch replacement, both of which necessitate reconstruction of the neck vessels, such as the brachiocephalic, left common carotid, and left subclavian arteries. In contrast, HAR was defined as a procedure that does not involve the reconstruction of neck vessels.

**Figure 1.**
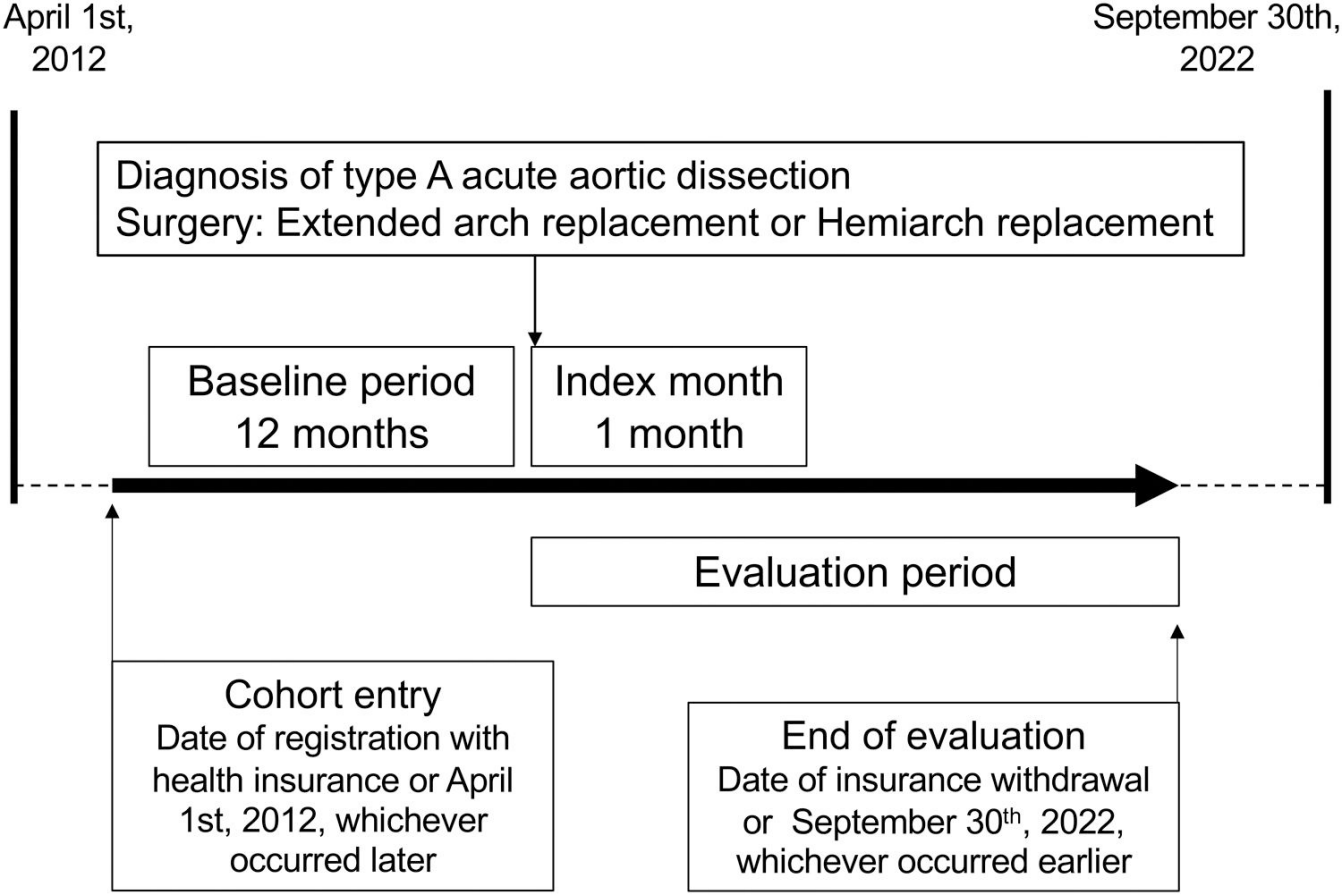
Study Design.

Patients were selected based emergency admission (index month) for a primary diagnosis of type A AAD with surgical procedure (EAR or HAR) between April 1, 2013 and September 30, 2022 (**Figure 1**). The baseline period for extraction of treatment histories for comorbidities was defined as the 12 months preceding the index month, and the evaluation period for detection of outcomes was defined as the period from the index month to the end of follow-up.

We excluded patients who had a delay of more than 1 month between the diagnosis of type A AAD and surgical intervention, as well as those who did not undergo surgery.

### Variables

Procedure selection was generally based on the location of the intimal tear: EAR was selected if the tear extended to the arch or beyond, while HAR was chosen if the entry was in the ascending aorta. In this study, the selection of each surgical procedure was based on the criteria determined by each participating institution. Additionally, several variables may have influenced the choice of surgical procedure and outcome, including age at diagnosis, sex, Charlson comorbidity index (**Table S1**), electrical frailty index (**Table S2**), residential area (classified as urban if the population exceeds 500 000), advanced treatment hospital (hospitals with ≥500 beds in Japan are defined as advanced treatment hospital, which are designated as tertiary care centres providing advanced medical services), past medical history (angiotensin-converting enzyme inhibitor/angiotensin II receptor blocker, calcium channel blocker, beta blocker, diuretic, antiplatelet drug, anticoagulant drug, and statin), whether the surgery was performed before or after 2020 (as the Japanese guidelines for aortic dissection were revised in 2020, which may have influenced the surgical decision-making), preoperative emergency procedures (endotracheal intubation and pericardiocentesis, which are reimbursable as procedural fees), and concomitant procedures (aortic valve and root surgery, mitral valve surgery, and coronary artery surgery) (**Table S3**).

### Outcomes

The primary outcome was defined as all-cause mortality. Mortality was determined based on withdrawal records in the insurance registry; specifically, patients whose withdrawal category was corded as “death” were defined as deaths. Secondary outcomes included reoperation for bleeding, aortic reoperation with artificial graft replacement (redo-EAR, redo-HAR [including root reconstruction], and descending aortic replacement), additional thoracic endovascular aortic repair (TEVAR), prolonged intensive care unit (ICU) stay (≥8 days), and total length of hospital stay.

### Statistical analysis

Categorical variables were expressed as numbers and percentages, while continuous variables were expressed as mean (standard deviations [SDs]) or median (interquartile ranges [IQRs]) based on the distribution.

To compare the effectiveness of EAR and HAR, propensity score matching (PSM) was employed to balance baseline characteristics between the 2 groups. The statistical significance of the outcomes after matching was evaluated using the Wilcoxon signed-rank test for continuous variables and the McNemar’s test for categorical variables. Propensity scores were calculated using logistic regression, incorporating the aforementioned covariates. Patients were matched in a 1:1 ratio using a greedy algorithm with a caliper width of 0.2 SD of the logit of the propensity score. Balance between the 2 groups was evaluated using standardized mean differences, with values <0.1 indicating adequate balance for each covariate. The fit of the model was assessed using the C-statistics and Hosmer-Lemeshow test to evaluate the discriminatory power of the propensity score model.

For the analysis of all-cause mortality, Kaplan-Meier survival curves were generated for each treatment group to estimate the survival probabilities over time, with day 0 defined as the day of admission. To account for the matched-pair design, the stratified log-rank test was used to compare survival distributions between the EAR and HAR groups. Furthermore, a Cox proportional hazards regression model with matched pairs included as a random effect was applied to examine the association between surgical procedures and mortality outcomes. The proportional hazards assumption was tested using Schoenfeld residuals.

Cumulative incidence functions (CIFs) were calculated for the incidence of aortic reoperation and TEVAR, accounting for death as a competing risk. Differences between groups were assessed using Gray’s test, which is suitable for comparing CIFs in the presence of competing risks. Fine-Gray subdistribution hazard models were employed to estimate the effect of the surgical procedures on these outcomes.

In the subgroup analysis, patients were stratified based on patient’s age (≥75 or <75 years) and surgical period (first half: before December 31, 2017; second half: from January 1, 2018 onward) to evaluate the potential impact of age and calendar time on the association between surgical procedure and outcomes. In a sensitivity analysis, we replicated the same analyses after excluding patients who died within 7 days postoperatively, a landmark analysis. This adjustment accounts for the possible influence of severe preoperative conditions, such as cardiac arrest and malperfusion, on the relationship between surgical methods and outcomes.

Statistical significance was set at *P* < .05. All statistical analyses were performed using JMP Pro version 17.0.0 (JMP Statistical Discovery LLC, Cary, NC, United States) and R version 4.2.2 (R Foundation for Statistical Computing, Vienna, Austria). We confirmed that there were no missing values in the dataset. Therefore, all analyses were conducted using the complete dataset, and no imputation or other specific methods for handling missing data were required.

## RESULTS

### Patients demographics

**Figure 2.**
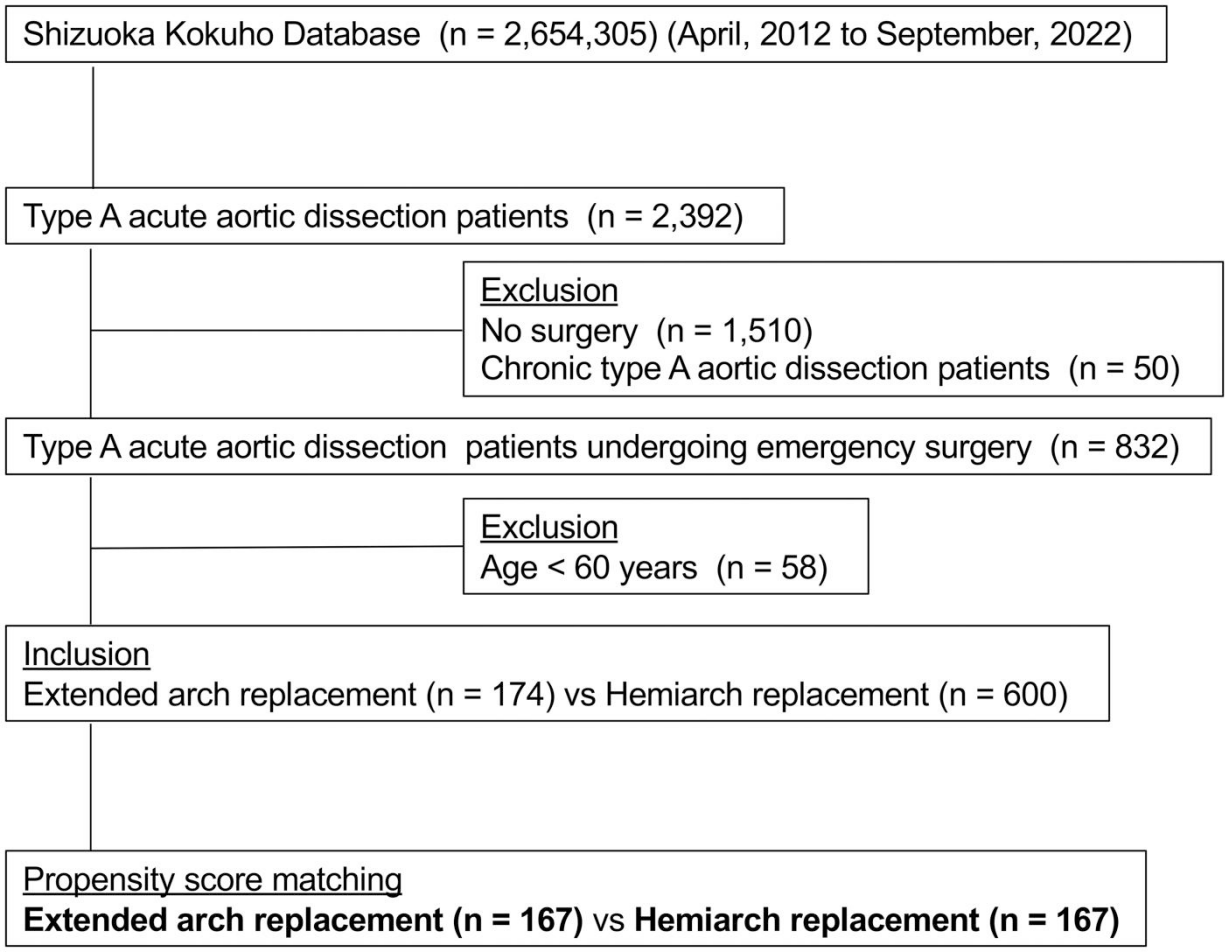
Flow Diagram of Study Cohort Enrolment.

Among the 2392 patients diagnosed with type A AAD, 1510 did not undergo surgery (**Figure 2**). Additionally, 50 patients were excluded due to a time interval of more than 1 month between diagnosis and surgery, and 58 patients were excluded because they were under 60 years of age. Finally, 774 patients were enrolled, of whom 174 underwent EAR and 600 underwent HAR. PSM resulted in 167 matched patient pairs (C-statistics 0.721 and Hosmer-Lemeshow test, *P* = .707).

Patient characteristics are shown in **Table 1**. Before PSM, patients in the EAR group were younger than those in the HAR group (73.3 [SD: 7.4] vs 75.9 [SD: 7.6]). Additionally, the EAR group had a larger proportion of males, urban residents, advanced treatment hospitals, and more cases after 2020 compared to the HAR group. After PSM, the baseline characteristics were comparable between the EAR and HAR groups. The median follow-up was 2.2 (IQR: 0.6-4.6) years.

**Table 1.** Patient Characteristics Before and After Propensity Score Matching

	Before propensity score matching			After propensity score matching		
	EAR	HAR	SMD	EAR	HAR	SMD
	*n* = 174	*n* = 600		*n* = 167	*n* = 167	
Age, mean (SD)	73.3 (7.4)	75.9 (7.6)	0.345	73.5 (7.5)	73.4 (7.4)	0.016
Male, *n* (%)	72 (41.4)	154 (25.7)	0.319	65 (38.9)	68 (40.7)	0.037
Urban, *n* (%)	85 (48.9)	184 (30.7)	0.363	78 (46.7)	77 (46.1)	0.012
Advanced treatment hospital, *n* (%)	133 (76.4)	332 (55.3)	0.497	126 (75.4)	125 (76.9)	0.014
EFI ≥ Moderate, *n* (%)	31 (17.8)	169 (28.2)	0.271	31 (18.6)	31 (18.6)	0.000
CCI ≥ High, *n* (%)	50 (28.7)	203 (33.8)	0.113	48 (28.7)	46 (27.5)	0.027
ACE-inhibitor/ARB, *n* (%)	55 (31.6)	207 (34.5)	0.062	51 (30.5)	50 (29.9)	0.013
Calcium channel blocker, *n* (%)	70 (40.2)	266 (44.3)	0.084	67 (40.1)	66 (39.5)	0.012
Beta blocker, *n* (%)	34 (19.4)	77 (12.8)	0.169	31 (18.6)	32 (19.2)	0.015
Diuretic, *n* (%)	12 (6.9)	74 (12.3)	0.215	12 (7.2)	15 (9.0)	0.071
Antiplatelet drug, *n* (%)	23 (13.2)	101 (16.8)	0.107	23 (13.8)	21 (12.6)	0.035
Anticoagulant drug, *n* (%)	12 (6.9)	38 (6.3)	0.022	11 (6.6)	13 (7.8)	0.047
Statin, *n* (%)	42 (24.1)	177 (29.5)	0.125	41 (24.6)	39 (23.4)	0.028
Emergency procedure,^a^ *n* (%)	40 (23.0)	112 (18.7)	0.030	38 (22.8)	39 (23.4)	0.029
After 2020, *n* (%)	68 (39.1)	169 (28.2)	0.224	65 (38.9)	62 (37.1)	0.037
Concomitant procedure,^b^ *n* (%)	18 (10.3)	72 (12.0)	0.053	17 (10.2)	17 (10.2)	0.000

a Emergency intubation and pericardiocentesis.

b Coronary artery bypass grafting, aortic and mitral valve surgery.

Abbreviations: ACE-inhibitor: angiotensin-converting enzyme inhibitor; ARB: angiotensin II receptor blocker; CCI: Charlson comorbidity index; EAR: extended arch replacement; EFI: electronic frailty index; HAR: hemiarch replacement; SD: standard deviation; SMD: standardized mean difference.

### Primary outcomes

**Figure 3.**
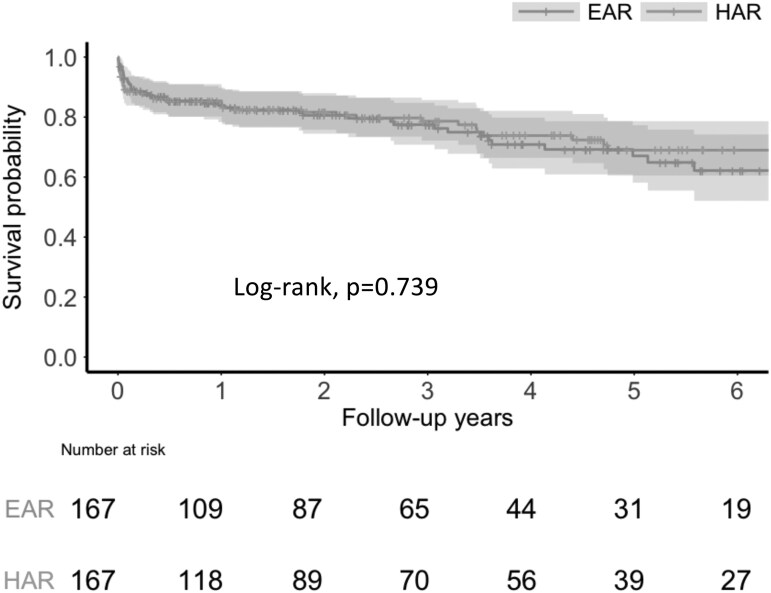
Kaplan-Meier Survival Curves for All-Cause Mortality in the Extended Arch and the Hemiarch Replacement Groups After Propensity Score Matching. The shaded areas represent 95% confidence intervals. Abbreviations: EAR: extended arch replacement; HAR: hemiarch replacement.

For the primary outcome, the Kaplan-Meier curves for all patients revealed no significant differences between the groups (**Figure 3**; log-rank test, *P* = .739). The Schoenfeld residual test confirmed that the proportional hazards assumption for the Cox model was satisfied (*P* = .330). Cox proportional hazards analysis also revealed no significant differences in all-cause mortality between the EAR and HAR groups (hazard ratio [HR]: 1.08, 95% confidence interval [CI]: 0.70-1.66).

In the subgroup analysis stratified by aged (patients age ≥75 or <75 years), there was no statistically significant difference in survival between the 2 groups in either stratum (**Figure S1A and B**). In an additional subgroup analysis that divided patients into the first and second halves of the surgical period, the survival curves between the EAR and HAR groups did not differ significantly (**Figure S2A and B**). However, a reversal in the survival trend was observed: the EAR group showed a lower survival rate in the first half, while the HAR group showed a lower survival rate in the second half.

### Secondary outcomes

**Figure 4.**
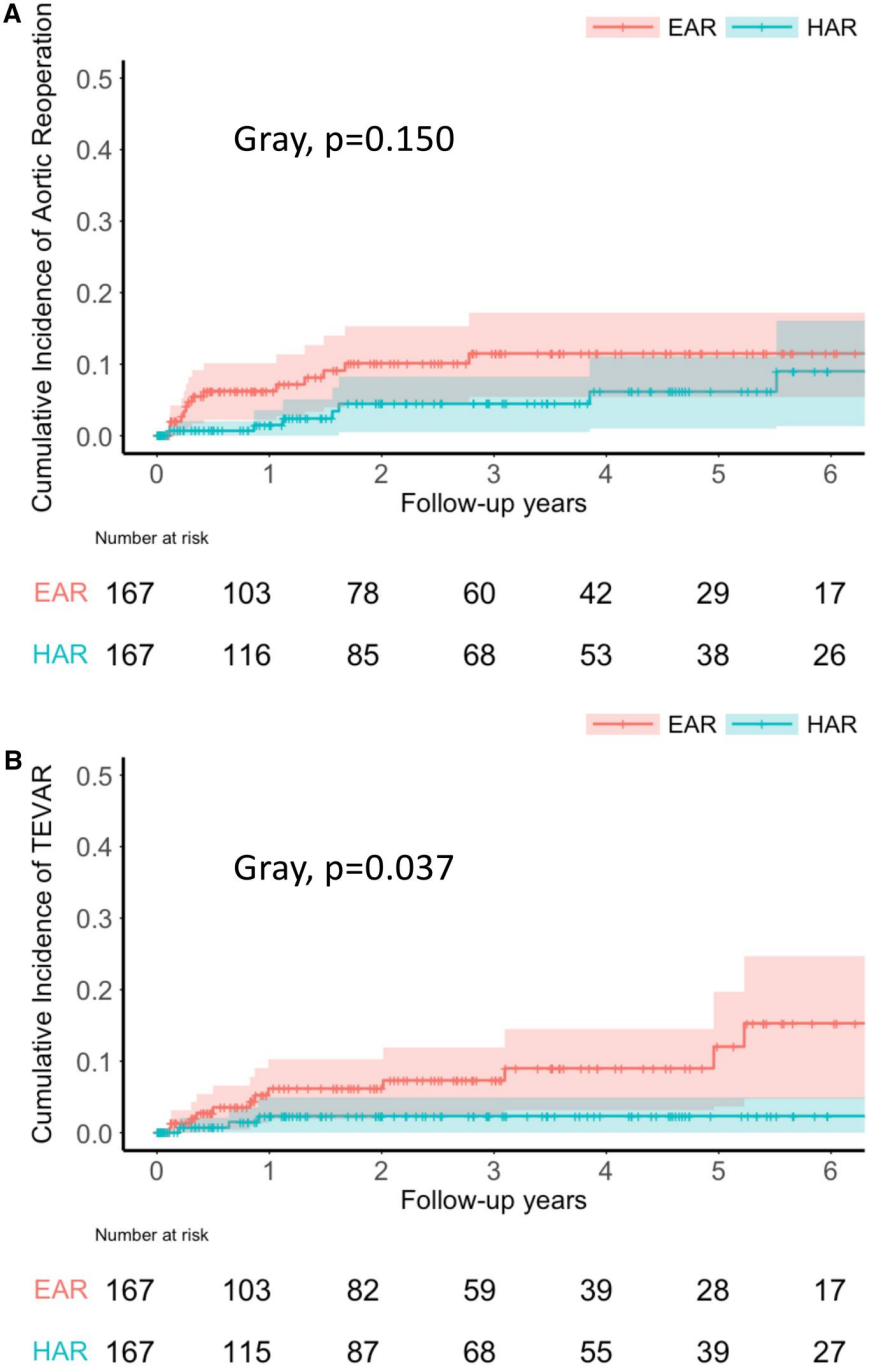
Cumulative incidence functions for aortic reoperation and thoracic endovascular aortic repair between the extended arch and the hemiarch replacement groups after propensity score matching. (A) Aortic reoperation (B) Thoracic endovascular aortic repar. The shaded areas represent 95% confidence intervals. Abbreviations: EAR: extended arch replacement; HAR: hemiarch replacement; TEVAR: thoracic endovascular aortic repair.

The CIF of aortic reoperation in the EAR group was no significant difference compared to the HAR group (**Figure 4A**; Gray test, *P* = .150), with a subdistribution HR (SHR) of 1.86 (95% CI: 0.80-4.34). In contrast, the CIF of TEVAR was significantly higher in the EAR group compared to the HAR group (**Figure 4B**; Gray test, *P* = .037), with a SHR of 3.27 (95% CI: 1.08-9.93). The aortic reoperations and TEVAR were performed at a median of 409 days (IQR: 178-580) and 234 days (IQR: 83.6-682.6), respectively. The proportion of patients requiring prolonged ICU stay was significantly higher in the EAR group than in the HAR group (EAR: 69 [41.3%] vs HAR: 49 [29.3%] patients, *P* = .029), as was the incidence of reoperation for bleeding (EAR: 20 [12.0%] vs HAR: 7 [4.2%] patients, *P* = .015). However, the length of hospital stay did not differ significantly between the 2 groups (EAR: 34.0 [IQR 20.0-73.0] vs HAR: 29.0 [18.0-60.0] days, *P* = .180).

### Sensitivity analysis

A total of 41 cases with early mortality within 7 days post-surgery were excluded from the landmark analysis and PSM resulted in 161 matched patient pairs. For the primary outcome, the Kaplan-Meier survival curve revealed no statistical difference between the EAR and HAR groups (log-rank test, *P* = .782, **Figure S3**). Cox proportional hazard analysis revealed no significant differences in all-cause mortality between the EAR and HAR groups (HR: 1.07, 95% CI: 0.68-1.68). For the secondary outcomes, the EAR group revealed higher proportions of prolonged ICU stay (67 [41.6%] vs 44 [27.3%], *P* = .010), reoperation for bleeding (20 [12.4%] vs 11 [6.2%], *P* = .042), TEVAR (SHR: 6.47, 95% CI: 1.44-29.0), and aortic reoperation (SHR: 2.73 95% CI: 0.98-7.49) compared to the HAR group.

## DISCUSSIONS

This study compared the postoperative outcomes of HAR and EAR in elderly patients with type A AAD. We found no significant differences in all-cause mortality between the 2 procedures in this population. However, EAR was associated with a higher incidence of reoperation for bleeding and additional TEVAR procedures and prolonged ICU stay. These findings underscore the importance of carefully selecting surgical approaches in elderly patients, given the potential for increased morbidity associated with EAR.

Our findings that postoperative outcomes did not differ significantly between EAR and HAR even in elderly patients is consistent with previous studies that mainly included younger populations.^6^^,^^7^ In clinical practice, surgeons tend to choose HAR for more severe or older patients, whereas EAR is often selected for less severe or younger patients. Variations in institutional expertise and surgeon experience, which could have influenced the choice of surgical procedure and outcomes, were not captured in this database and may have introduced centre-related bias. However, these factors were partially accounted for using hospital size as a proxy. The results remained unchanged after reanalysis including this variable, indicating that centre-related bias was unlikely to have substantially influenced the findings.

Critically ill patients, especially those with cardiopulmonary arrest or malperfusion, have higher early postoperative mortality compared to patients without such complications.^8^ Postoperative outcomes were more strongly influenced by the severity of type A AAD than by the surgical procedure itself.^9^ In this study, the preoperative severity was not accurately reflected. Previous study has shown that severe preoperative conditions often result in early postoperative mortality, particularly within the first 7 days.^10^ Following this precedent, a sensitivity analysis was conducted using a landmark analysis that excluded cases of mortality within 7 days postoperatively, yielding findings consistent with those of the primary analysis. Although excluding severe cases helps reduce survivor bias, this may not fully account for all factors affecting outcomes. Furthermore, the higher survival rate of the EAR group compared to the HAR group observed in the second half of inclusion period subgroup analysis may be attributable to advancements in new medical devices, such as the impact of frozen elephant trunk (FET) procedure, which constitute unmeasured confounders. These differences in patient characteristics could influence postoperative outcomes and complicate direct comparisons of EAR and HAR effectiveness. However, upon examining the progression of survival curves, neither the EAR nor HAR group showed substantial differences in long-term survival rates, which is inconsistent with previous studies reporting higher in-hospital mortality associated with EAR among the eldery.^11^^,^^12^ This lack of difference in outcomes may be attributable to on-site clinicians at each institution appropriately selecting surgical procedures based on individual patient characteristics.

In this study, we defined the elderly population as those aged ≥60 years. Although this threshold is lower than the conventional cut-off of 70-75 years commonly used in cardiovascular surgery studies, it reflects the sociocultural and clinical context in Japan, where individuals aged 60 years or older are often considered elderly and constitute a large proportion of surgical candidates. In the present study, patients aged 60-69 years accounted for approximately one-third of the total cohort. Therefore, our results mainly represent the “young-old” population, and outcomes might differ in older age groups.

Our study found that the EAR group was more likely to undergo reoperation for bleeding and TEVAR than the HAR group, while the incidence of aortic reoperations was not significantly different between the groups. EAR is a more technically demanding procedure than HAR and tends to require longer periods of cardiopulmonary bypass, potentially increasing the risk of early reoperation for bleeding.^7^^,^^12^ Furthermore, the higher proportion of patients with prolonged ICU stay in the EAR group reflects the greater complexity of postoperative management associated with this procedure.

In our study, aortic reoperations and TEVAR were mainly performed in the late phase (median 409 days and 234 days, respectively) for aortic complications or aneurysmal changes, rather than as planned early staged procedures. Data regarding the location of residual tears and the degree of aortic dilatation were not available; therefore, their potential impact on long-term outcomes remains uncertain. The lack of a significant difference in aortic reoperation rates between the both groups may be explained by possibility that reoperations were deferred in some patients because of advanced age or poor general health conditions. Although, the median follow-up duration of 2.2 years may be relatively short to fully captured very late aortic events, previous studies have reported that most late aortic complications after HAR tend to occur within approximately 2 to 5 years postoperatively.^13–15^ Therefore, our follow-up period likely covered the early phase during which the majority of these events are observed, while longer-term and imaging-based evaluations remains warranted.

The increased number of TEVAR procedures in the EAR group might be due to the structurally easier additional treatment with TEVAR. Notably, second-stage TEVAR management of residual aortic dissection after initial FET technique induced significant remodelling of the entire aorta.^16^ In Japan, the 2020 revision of the aortic dissection guidelines suggested that total arch replacement with FET may contribute to less invasive procedures^17^ and is likely to be increasingly chosen as the procedure for type A AAD. According to the 2023 annual report on the Japanese Association for Thoracic Surgery, total arch replacement accounted for 32.3% of surgeries for type A AAD, and FET technique was used in 93.3% of those cases.^18^ The better survival of the EAR group compared to the HAR group in the second half of surgical period may have been influenced by introduction of FET.

The present study has several limitations. First, the SKDB lacks detailed clinical information, including the severity of the aortic dissection (such as cardiopulmonary arrest, malperfusion and rupture), anatomical details (such as the location of the intimal tear and the extent of dissection), imaging findings (computed tomography, magnetic resonance imaging, and echocardiography), and specific surgical parameters (such as operation time, cardiopulmonary bypass time, cerebral protection strategies). Therefore, residual confounding by indication or underlying anatomical complexity cannot be completely ruled out. Second, indications for surgery, surgical techniques, and postoperative care may have varied across institutions and were not standardized. Third, the long inclusion period may have influenced the results, as both treatment strategies and surgical techniques could have evolved over time, particularly after the introduction of the FET technique. Fourth, this study was conducted within the Japanese healthcare system, where differences in device availability, reimbursement policies, and surgical practice—such as the FET technique—may influence treatment selection and outcomes. Therefore, caution is warranted when generalizing our findings to countries with different healthcare structures or surgical practice.

## CONCLUSION

The present study found no statistically significant difference in postoperative mortality between EAR and HAR in elderly patients with type A AAD. While the wide CI suggests uncertainty regarding a true survival difference, EAR was associated with higher postoperative morbidity, including reoperation for bleeding and prolonged ICU stay. These findings suggest that the choice of surgical procedure in elderly patients should be made with careful consideration of both potential benefits and perioperative risks.

## Supplementary Material

ivag017_Supplementary_Data

## Data Availability

According to the terms of Shizuoka Prefecture’s data use agreement with local insures, the analysed data cannot be provided to readers by the authors. Researchers interested in accessing this dataset may apply to Shizuoka Prefecture to request access. Please contact the staff of Shizuoka Graduate University of Public Health (e-mail: info@s-sph.ac.jp).
