# Supplementary material for: Comparison of Extended Arch Versus Hemiarch Replacement in Elderly Patients With Type A Aortic Dissection: The Shizuoka Kokuho Database
Source: Interdiscip Cardiovasc Thorac Surg. 2026 Jan 10;41(1):ivag017. doi: 10.1093/icvts/ivag017 (PMC12836426; doi:10.1093/icvts/ivag017)
Supplement: ivag017_Supplementary_Data [file ivag017_supplementary_data.zip › Supplemental materials.docx]

**Figure legend**

**Figure S1:** Kaplan-Meier survival curves for all-cause mortality comparing the extended arch and the hemiarch replacement groups after propensity score matching in (A) patients aged ≥75 years and (B) patients aged <75 years. The shaded areas represent 95% confidence intervals.

EAR, extended arch replacement; HAR, hemiarch replacement

**Figure S2:** Kaplan-Meier survival curves for all-cause mortality comparing the extended arch and the hemiarch replacement groups after propensity score matching in (A) the first (~2027/12/31) and (B) second (2018/01/01~) halves of the surgical period. The shaded areas represent 95% confidence intervals.

EAR, extended arch replacement; HAR, hemiarch replacement

**Figure S3:** Kaplan-Meier survival curves for all-cause mortality in the extended arch and the hemiarch replacement groups, following exclusion of early deaths (within 7 days postoperatively) and propensity score matching. The shaded areas represent 95% confidence intervals.

EAR, extended arch replacement; HAR, hemiarch replacement.

**Table S1. List of diseases, codes and scores for Charlson comorbidity index**

| Disease | International Classification of Diseases, 10^th^ revision | Score |
| --- | --- | --- |
| Myocardial infarction | I21, I22, I252 | 1 |
| Congestive heart failure | I099, I110, I130, I132, I255, I420, I425, I426, I427, I428, I429, I43, I50, P290 | 1 |
| Peripheral vascular disease | I70, I71, I731, I738, I739, I771, I790, I792, K551, K558, K559, Z958, Z959 | 1 |
| Cerebrovascular disease | G45, G46, H340, I60, I61, I62, I63, I64, I65, I66, I67, I68, I69 | 1 |
| Dementia | F00, F01, F02, F03, F051, G30, G311 | 1 |
| Chronic pulmonary disease | I278, I279, J40, J41, J42, J43, J44, J45, J46, J47, J60, J61, J62, J63, J64, J65, J66, J67, J684, J701, J703 | 1 |
| Rheumatic disease | M05, M06, M315, M32, M33, M34, M351, M353, M360 | 1 |
| Peptic ulcer disease | K25, K26, K27, K28 | 1 |
| Mild liver disease | B18, K700, K701, K702, K703, K709, K713, K714, K715, K717, K73, K74, K760, K762, K763, K764, K768, K769, Z944 | 1 |
| Diabetes without chronic complication | E100, E101, E106, E108, E109, E110, E111, E116, E118, E119, E120, E121, E126, E128, E129, E130, E131, E136, E138, E139, E140, E141, E146, E148, E149 | 1 |
| Diabetes with chronic complication | E102, E103, E104, E105, E107, E112, E113, E114, E115, E117, E122, E123, E124, E125, E127, E132, E133, E134, E135, E137, E142, E143, E144, E145, E147 | 2 |
| Hemiplegia or paraplegia | G041, G114, G801, G802, G81, G82, G830, G831, G832, G833, G834, G839 | 2 |
| Renal disease | I120, I131, N032, N033, N034, N035, N036, N037, N052, N053, N054, N055, N056, N057, N18, N19, N250, Z490, Z491, Z492, Z940, Z992 | 2 |
| Any malignancy, including lymphoma and leukemia, except malignant neoplasm of skin | C00, C01, C02, C03, C04, C05, C06, C07, C08, C09, C10, C11, C12, C13, C14, C15, C16, C17, C18, C19, C20, C21, C22, C23, C24, C25, C26, C30, C31, C32, C33, C34, C37, C38, C39, C40, C41, C43, C45, C46, C47, C48, C49, C50, C51, C52, C53, C54, C55, C56, C57, C58, C60, C61, C62, C63, C64, C65, C66, C67, C68, C69, C70, C71, C72, C73, C74, C75, C76, C81, C82, C83, C84, C85, C88, C90, C91, C92, C93, C94, C95, C96, C97 | 2 |
| Moderate or severe liver disease | I850, I859, I864, I982, K704, K711, K721, K729, K765, K766, K767 | 3 |
| AIDS / HIV | B20, B21, B22, B24 | 6 |
| Metastatic solid tumor | C77, C78, C79, C80 | 6 |

AIDS, acquired immune deficiency syndrome; HIV, human immunodeficiency virus.

**Table S2. List of diseases and codes for electrical frailty index**

| Disease | | International Classification of Diseases, 10^th^ revision | |
| --- | --- | --- | --- |
| Activity limitation | | R26, S78, S88, S98, T13.6, Y83, Z99.3, G11, G81, G82, G83, M62 | |
| Anemia and hematinic deficiency | | D50, D51, D52, D53, D64 | |
| Arthritis | | M05, M06, M07, M09, M10, M11, M12, M13, M15, M16, M17, M18, M19, M31.5, M32, M33, M34, M35, M36 | |
| Atrial fibrillation | | I44, I48, I49 | |
| Cerebrovascular disease | | G45, G46, I6, H34 | |
| Chronic kidney disease | | I12, I13, N01, N03, N05, N07, N08, N18, N19, N25, I77 | |
| Diabetes | | E10.9, E11.9, E12.9, E13.9, E14.9 | |
| Dizziness | | I95, R55, R42, E86, H81, H82, H83 | |
| Dyspnea | | R06 | |
| Falls | | Not available | |
| Foot problems | | B353, G575, G576, L60, M201, M202, M203, M204, M205, M206, M213, M214, M215, M216, M722, M766, M773, M775, S90, S91, S92, S93, S94, S96, S97, S99, Q66 | |
| Fragility fracture | | M484, S22, S32, S33, S42, S43, S62, S72, S73, M485, M800, M808, M843, M847, S02, S12, S52, S82, S92 | |
| Hearing impairment | | H833, H90, H91, G960, H60, H61, H62, H71, H73, H74, H92, H93, I11, I13 | |
| Heart failure | | I26.0, I27, I42, I43, I50, I51, I09, I255 | |
| Heart valve disease | | I05, I06, I07, I34, I35, I36, I37, I390, I391, I392, I393, I394, A520, I091, I098, I38, Q230, Q231, Q232, Q233 | |
| Housebound | | R40, Z50, Z74, Z75.5 | |
| Hypertension | | I10, I11, I12, I13, H350 | |
| Hypotension/syncope | | I95, R55, R52, E86 | |
| Ischemic heart disease | | I20, I21, I22, I23, I24, I25 | |
| Memory and cognitive problems | | F00, F01, F02, F03, F04, F05, F06.7, G30, G31, R41, R54, F2, F3, F41, R44, R45 | |
| Mobility and transfer problems | | R26, S78, S88, S98, T13.6, Y83, Z99.3, G11, G81, G82, G83, M62 | |
| Osteoporosis | | M80, M81, M82 | |
| Parkinsonism and tremor | | G122, G20, G21, G22, G23, G25, G26, G32, G35, R25 | |
| Peptic ulcer | | K21, K25, K26, K27, K28, K29, R12 | |
| Peripheral vascular disease | | I65, I70, I71, I82, I73, I771, K551, K558, K559, R02, Z958, Z959, I790, I792, Z958 | |
| Polypharmacy | | ≥ 5 drugs prescriptions for a total of ≥12 months during the baseline period | |
| Requirement for care | | R40 | |
| Respiratory disease | | J45, J46, J40, J41, J42, J43, J44, J47, J60, J61, J62, J63, J64, J65, J67, J684, J70, J13, J14, J15, J16, J18, J22, J20, J90, J961, J980 | |
| Skin ulcer | | I83, I98, L03, L08, L89, L97, L984 | |
| Sleep disturbance | | G47, F51 | |
| Social vulnerability | | F1, R460, R468, Z59, Z60, Z63, Z73 | |
| Thyroid disease | | E00, E01, E03, E04, E05, E06, E079, E890, R946 | |
| Urinary incontinence | | N31, N393, N394, R15, R32, Z466 | |
| Urinary system disease | | N30, N34, N39.0, N39.8, N39.9, R31, R33, T835 | |
| Visual impairment | | H25, H28, H35, H40, H43, H53, H54 | |
| Weight loss and anorexia | | E41, E43, E44, E46, E53, E55, E66, E83, E87, R53, R628, R63, R64, F500, F501, F508, F509 | |
|  | |  |  |

**Table S3. List of procedures and bill codes**

| Procedure | Bill code |
| --- | --- |
| Hemiarch replacement | 150245010 |
| Extended arch replacement | 150150010, 150275910, 150381550, 150381950 |
| Hemiarch or Extended arch replacement with AVR / Bentall / VSRR | 150244910, 150359510, 150359610, 150359710, 150381650, 150359810, 150381750, 150359910, 150381850 |
| Mitral Valve repair / replacement | 150141410, 150141610, 150141010, 150279510 |
| Coronary artery bypass grafting | 150145910, 150146010 |
| Emergency intubation | 140009010 |
| Pericardiocentesis | 140010510 |
| ICU management ≥ 8 days | 190174510, 190246610, 190174710, 190246810, 190116410, 190247010, 190139910, 190247210, 193010110, 193010310, 193501710, 193507610, 193511810, 193512010, 193301510, 193307310, 193310310, 193310510 |
| Reoperation for bleeding | 150140510, 150138410, 150138210, 150124710 |
| TEVAR | 150301310 |
| AVR, Aortic valve replacement; ICU, intensive care unit, TEVAR, thoracic endovascular aortic repair; VSRR, valve sparing root replacement. | |
