# Supplementary figures and images for: Comparison of Extended Arch Versus Hemiarch Replacement in Elderly Patients With Type A Aortic Dissection: The Shizuoka Kokuho Database
Source: Interdiscip Cardiovasc Thorac Surg. 2026 Jan 10;41(1):ivag017. doi: 10.1093/icvts/ivag017 (PMC12836426; doi:10.1093/icvts/ivag017)

(A)

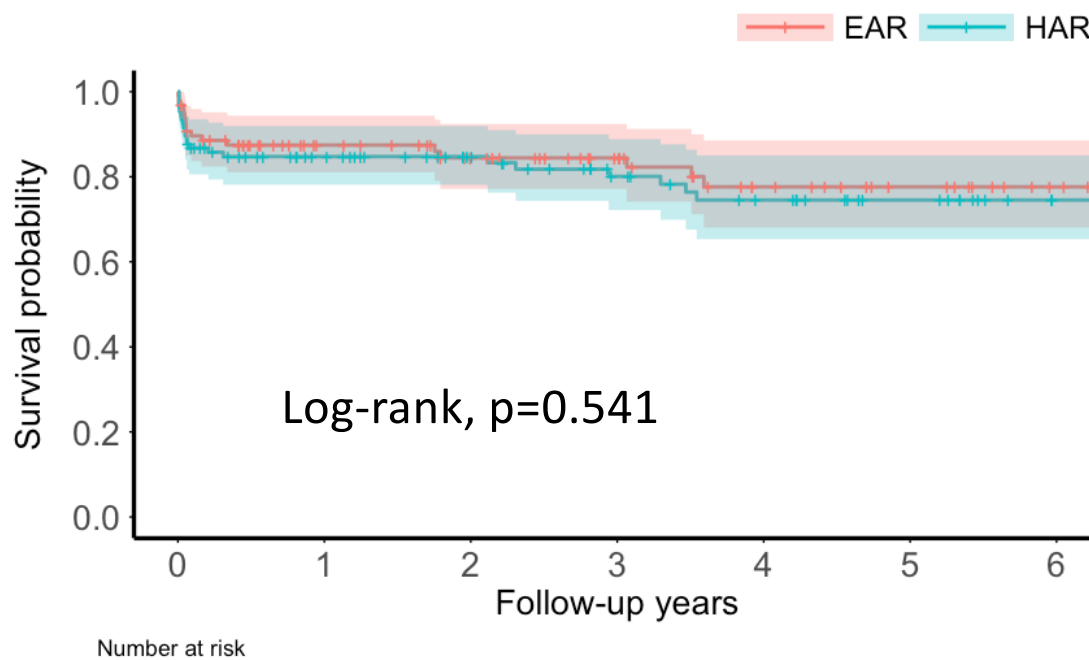

|     |     |    |    |    |    |    |    |
|-----|-----|----|----|----|----|----|----|
| EAR | 97  | 64 | 53 | 42 | 28 | 21 | 13 |
| HAR | 106 | 73 | 59 | 46 | 38 | 30 | 20 |

(B)

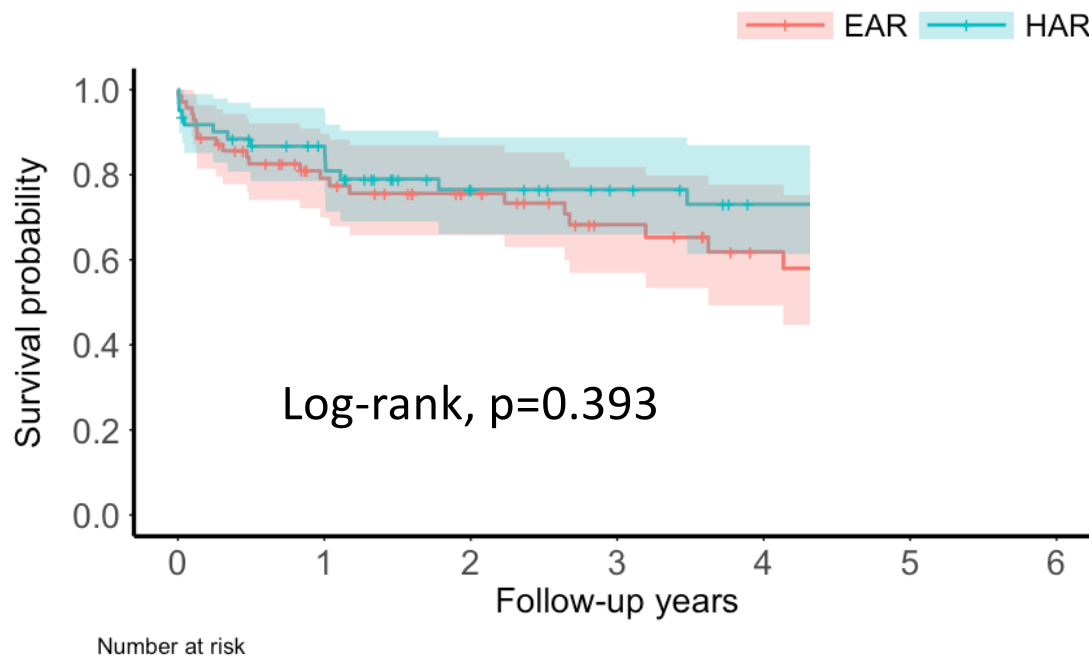

|     |    |    |    |    |    |
|-----|----|----|----|----|----|
| EAR | 70 | 45 | 34 | 23 | 16 |
| HAR | 61 | 45 | 30 | 24 | 18 |

Supplement: ivag017_Supplementary_Data [file ivag017_supplementary_data.zip › figureS1_revision.pdf]

(A)

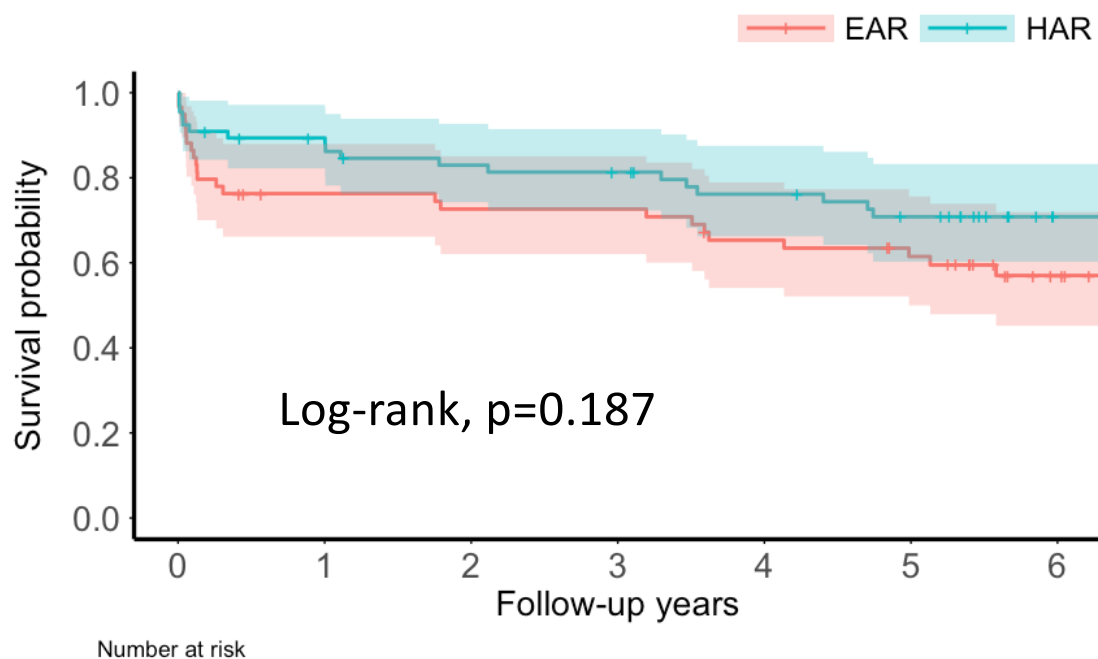

(B)

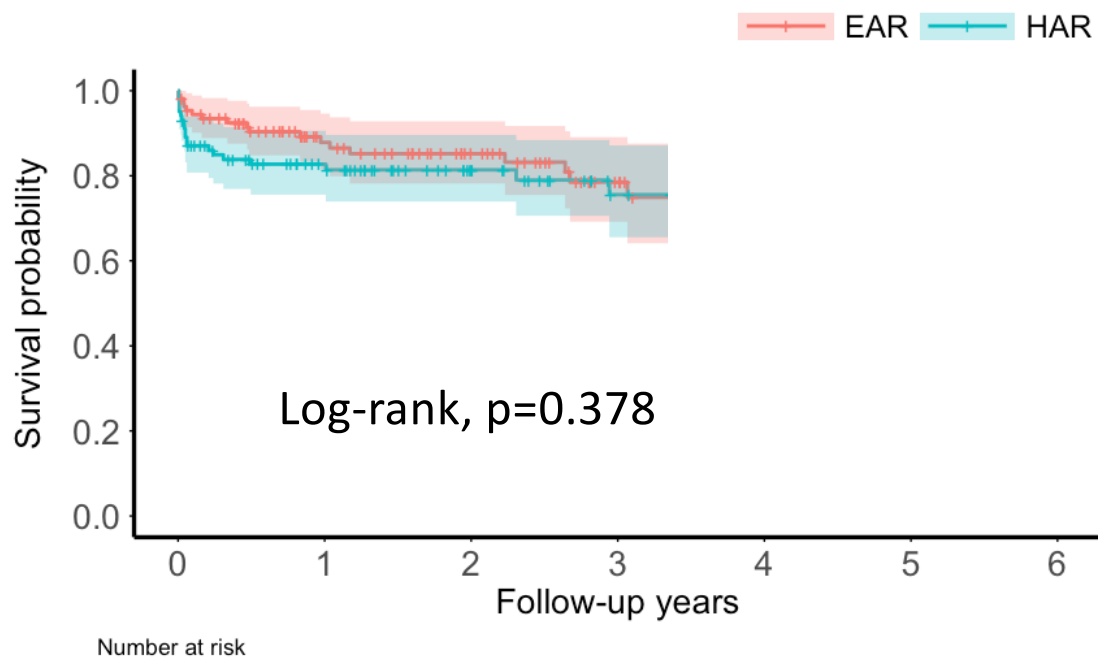

Supplement: ivag017_Supplementary_Data [file ivag017_supplementary_data.zip › FigureS2_revision.pdf]

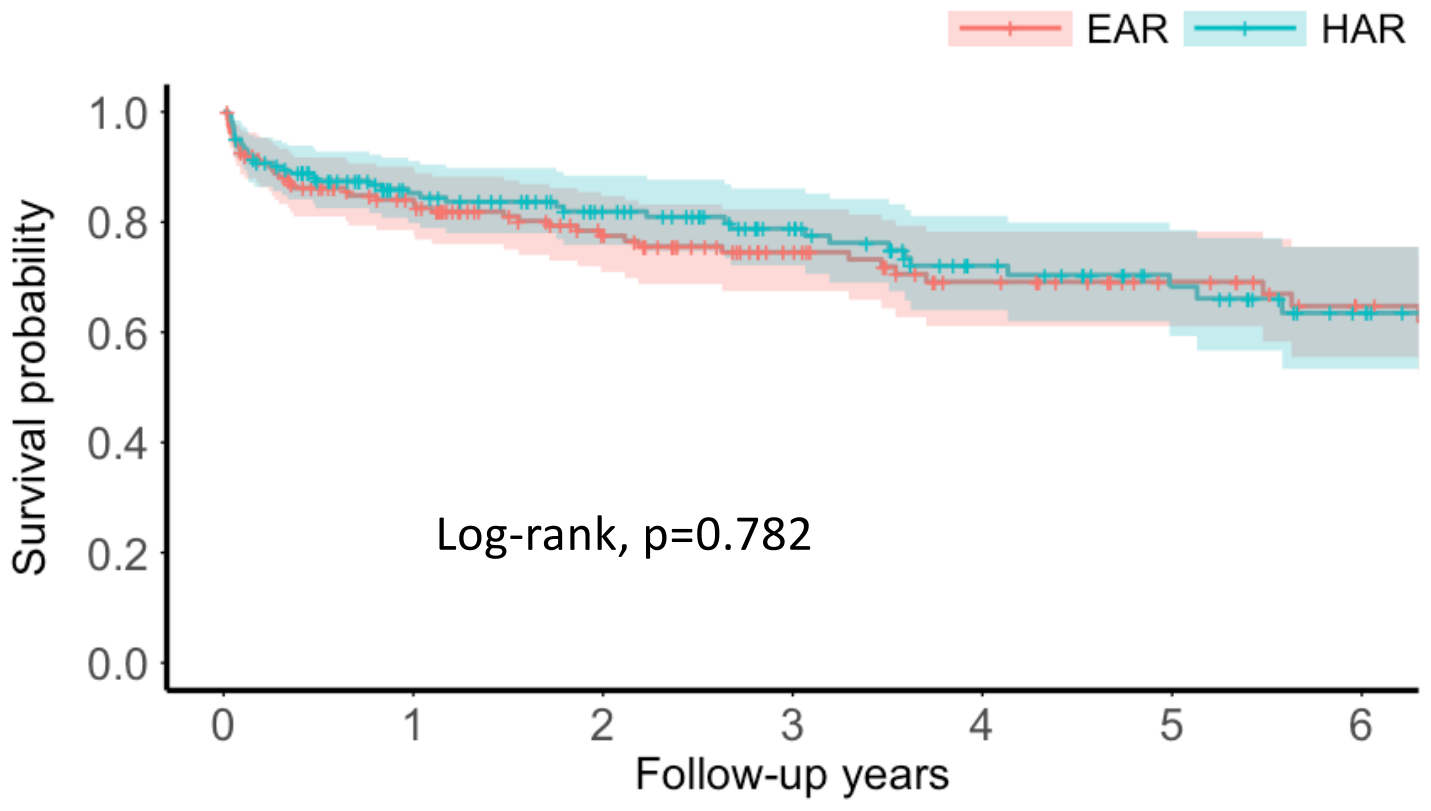

Number at risk

|     |     |     |    |    |    |    |    |
|-----|-----|-----|----|----|----|----|----|
| EAR | 163 | 115 | 83 | 61 | 46 | 36 | 26 |
| HAR | 163 | 109 | 87 | 65 | 44 | 32 | 20 |

Supplement: ivag017_Supplementary_Data [file ivag017_supplementary_data.zip › FigureS3_revision.pdf]
